# Supplementary material for: KIF1C activates and extends dynein movement through the FHF cargo adapter
Source: Nat Struct Mol Biol. 2025 Jan 2;32(4):756–66. doi: 10.1038/s41594-024-01418-z (PMC11996680; doi:10.1038/s41594-024-01418-z)
Supplement: Supplementary file 1 — Reporting Summary [file 41594_2024_1418_MOESM1_ESM.pdf]

nature portfolio

Corresponding author(s): Andrew P. Carter and Anne Straube

Last updated by author(s): 30/05/2024

Reporting Summary

Nature Portfolio wishes to improve the reproducibility of the work that we publish. This form provides structure for consistency and transparency in reporting. For further information on Nature Portfolio policies, see our Editorial Policies and the Editorial Policy Checklist.

Statistics

For all statistical analyses, confirm that the following items are present in the figure legend, table legend, main text, or Methods section.

n/a	Confirmed	
☐	☒	The exact sample size (*n*) for each experimental group/condition, given as a discrete number and unit of measurement
☐	☒	A statement on whether measurements were taken from distinct samples or whether the same sample was measured repeatedly
☐	☒	The statistical test(s) used AND whether they are one- or two-sided<br>*Only common tests should be described solely by name; describe more complex techniques in the Methods section.*
☒	☐	A description of all covariates tested
☒	☐	A description of any assumptions or corrections, such as tests of normality and adjustment for multiple comparisons
☐	☒	A full description of the statistical parameters including central tendency (e.g. means) or other basic estimates (e.g. regression coefficient) AND variation (e.g. standard deviation) or associated estimates of uncertainty (e.g. confidence intervals)
☐	☒	For null hypothesis testing, the test statistic (e.g. *F*, *t*, *r*) with confidence intervals, effect sizes, degrees of freedom and *P* value noted<br>*Give P values as exact values whenever suitable.*
☒	☐	For Bayesian analysis, information on the choice of priors and Markov chain Monte Carlo settings
☒	☐	For hierarchical and complex designs, identification of the appropriate level for tests and full reporting of outcomes
☒	☐	Estimates of effect sizes (e.g. Cohen's *d*, Pearson's *r*), indicating how they were calculated

Our web collection on statistics for biologists contains articles on many of the points above.

Software and code

Policy information about availability of computer code

Data collection	1. Cryo-EM data collection: EPU 2.6.1<br>2. Single molecule TIRF data collection: Micromanager v1.4 and xCellence
Data analysis	1. Image processing: RELION v4.0 and wrappers within: MotionCor2, CTFFIND4<br>2. Particle picking: crYOLO 1.7.5<br>3. Model building and refinement: Coot 0.93 and PHENIX 1.14 and 1.20<br>4. Model and map analysis: UCSF Chimera 1.14 and UCSF Chimera X 1.4<br>5. Structure prediction: AlphaFold2, Colabfold 1.5.2 and AlphaScreen (https://github.com/sami-chaaban/alphascreen)<br>6. Star file mining: StarParser 1.38 (https://github.com/sami-chaaban/starparser)<br>7. Mass photometry: Refeyn AcquireMP and DiscoverMP, v2.3<br>8. Kymograph analysis: FiJi v2.9.0<br>9. Graphs and statistics: Prism v9.0.0 and python custom scripts<br>10. Sequence alignments: Jalview 9.0.5<br>11. Conservation analysis: ConSurf web server<br>12. Adobe Illustrator 2023 and 2024 (Adobe)

For manuscripts utilizing custom algorithms or software that are central to the research but not yet described in published literature, software must be made available to editors and reviewers. We strongly encourage code deposition in a community repository (e.g. GitHub). See the Nature Portfolio guidelines for submitting code & software for further information.

Data

Policy information about availability of data

All manuscripts must include a data availability statement. This statement should provide the following information, where applicable:

- Accession codes, unique identifiers, or web links for publicly available datasets
- A description of any restrictions on data availability
- For clinical datasets or third party data, please ensure that the statement adheres to our policy

Atomic coordinates and cryo-EM maps have been deposited in the protein data bank (PDB) and Electron Microscopy Data Bank (EMDB) under the accession codes EMD-18302 and PDB 8QAT for FHF structure and EMD-18303 for FHF + KIF1C stalk. All gel filtration, SDS-PAGE, crosslinking mass spectrometry, single molecule microscopy and AlphaFold2 raw data and associated analysis tables are deposited to Zenodo (10.5281/zenodo.10949991 and 10.5281/zenodo.11360634).

Research involving human participants, their data, or biological material

Policy information about studies with human participants or human data. See also policy information about sex, gender (identity/presentation), and sexual orientation and race, ethnicity and racism.

Reporting on sex and gender	n/a
Reporting on race, ethnicity, or other socially relevant groupings	n/a
Population characteristics	n/a
Recruitment	n/a
Ethics oversight	n/a

Note that full information on the approval of the study protocol must also be provided in the manuscript.

Field-specific reporting

Please select the one below that is the best fit for your research. If you are not sure, read the appropriate sections before making your selection.

☒ Life sciences ☐ Behavioural & social sciences ☐ Ecological, evolutionary & environmental sciences

For a reference copy of the document with all sections, see nature.com/documents/nr-reporting-summary-flat.pdf

Life sciences study design

All studies must disclose on these points even when the disclosure is negative.

Sample size	The sample size for cryo-EM was not pre-determined and depended on data collection of a first dataset (ranging between 5,000-26,000 movies depending on the time allocated on the electron microscope). If resulting structure did not yield side-chain resolution and/or an isotropic cryo-EM density then additional datasets were collected and combined. This strategy improved the resolution obtained for the FHF structure and provided additional missing views to overcome anisotropy. If adding the new data did not improve the density significantly, as observed with the FHF-KIF1C stalk datasets, then no further data were collected and cryo-EM analysis was combined with other methods to improve assignment of protein domains. The sample size for single molecule TIRF data in Figures 2 and 7 was not pre-determined. Several technical replicates were performed on three different experiment days and all usable data pooled for each day. Power analysis tests were performed for single molecule TIRF data in Figure 4 to determine how many technical replicates to perform. 15 microtubules per technical n for FHF-containing samples was determined to be feasible based on the average concentration of microtubules in the field of view.
Data exclusions	Exclusions for cryo-EM analysis included ice "particles", carbon edges, junk/non-averaged protein density and low-resolution/broken density classes during particle sorting stages (2D and 3D classification). For analysis of TIRF data in Figure 4, any events that were stationary or moved <1.2s duration or <500 nm were discounted and not classed in the processive event or run length classification. For analysis of TIRF data in Figures 1, 2 and 7, data were only analysed from single microtubules that showed exclusively unidirectional runs after KIF1C-GFP flow-in. Runs were defined as detailed in the methods.
Replication	Cryo-EM data findings were replicated through the collection of 5 separate and distinct datasets (from different FHF preparations), revealing an identical architecture and organization of FHF. FHF-containing TIRF datasets were repeated at least four times (often using proteins from distinct purifications [biological replicates]), each time showing the same trend of results.
Randomization	For calculation of the gold-standard FSC, cryo-EM particles were randomly split into two halves using RELION.
Blinding	Blinding is not relevant for structural studies here as grouping is not applicable to image processing. For FHF-containing single molecule studies, blinding would not be appropriate as the conditions showed a clear phenotype that would be straightforward to distinguish even in the absence of sample labelling. For TIRF assays shown in Figures 1, 2 and 7, all single microtubules were included in kymograph analysis and

all motors were traced in kymographs. Filtering of runs and measurements was extracted via scripts in an unbiased manner, making blinding obsolete.

Reporting for specific materials, systems and methods

We require information from authors about some types of materials, experimental systems and methods used in many studies. Here, indicate whether each material, system or method listed is relevant to your study. If you are not sure if a list item applies to your research, read the appropriate section before selecting a response.

Materials & experimental systems

n/a	Involved in the study
☒	☐ Antibodies
☐	☒ Eukaryotic cell lines
☒	☐ Palaeontology and archaeology
☒	☐ Animals and other organisms
☒	☐ Clinical data
☒	☐ Dual use research of concern
☒	☐ Plants

Methods

n/a	Involved in the study
☒	☐ ChIP-seq
☒	☐ Flow cytometry
☒	☐ MRI-based neuroimaging

Eukaryotic cell lines

Policy information about cell lines and Sex and Gender in Research

Cell line source(s)	Sf9 cells were used for expression and purification of HOOK3, FHF, dynein, KIF1C-GFP and Lis1 (ThermoFisher Scientific cat. no. 11496015). Dynactin was purified from pig brains. KIF1C stalk constructs were expressed and purified from E.coli SoluBL21 cells (Bio Cat GMB Cat. No. C700200-GL).
Authentication	No cell line authentication was used.
Mycoplasma contamination	Cells were not tested for mycoplasma contamination.
Commonly misidentified lines (See ICLAC register)	No commonly misidentified cell lines were used.

Plants

Seed stocks	n/a
Novel plant genotypes	n/a
Authentication	n/a
