# Supplementary figures and images for: KIF1C activates and extends dynein movement through the FHF cargo adapter
Source: Nat Struct Mol Biol. 2025 Jan 2;32(4):756–66. doi: 10.1038/s41594-024-01418-z (PMC11996680; doi:10.1038/s41594-024-01418-z)

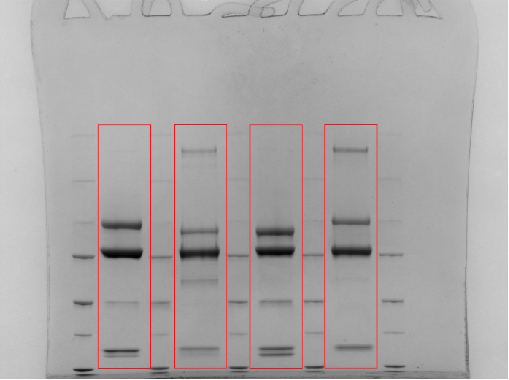

Supplement: Supplementary file 5 — Unprocessed coomassie stained SDS–PAGE gel of Fig. 3f. [file 41594_2024_1418_MOESM5_ESM.jpg]

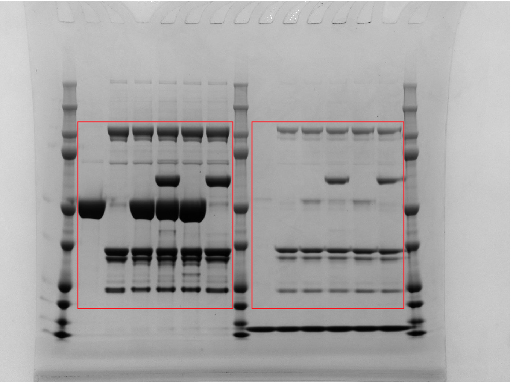

Supplement: Supplementary file 6 — Unprocessed coomassie stained SDS–PAGE gel of Fig. 5g. [file 41594_2024_1418_MOESM6_ESM.jpg]

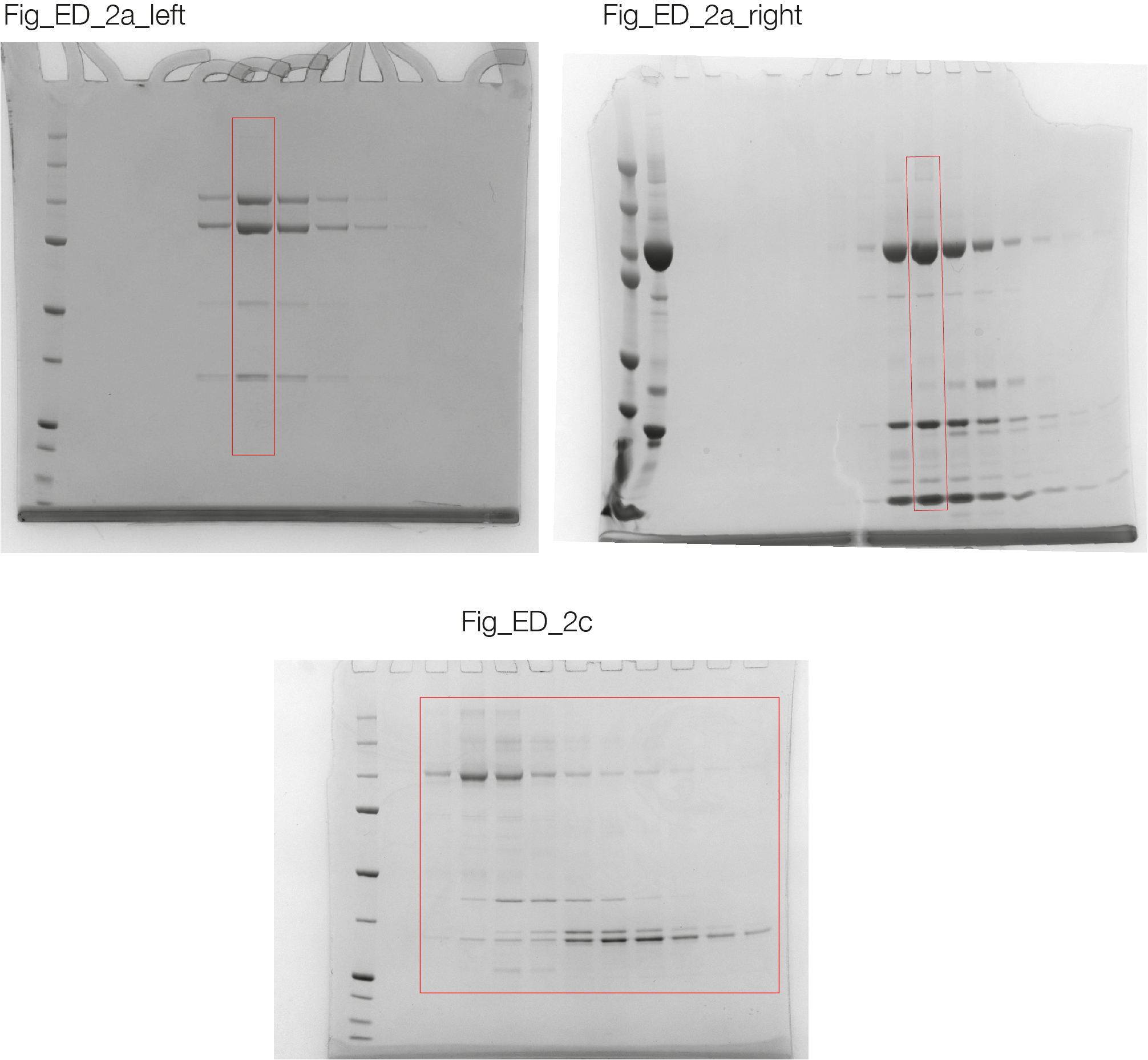

Supplement: Supplementary file 7 — Unprocessed coomassie stained SDS–PAGE gels of Extended Data Fig. 2a,c. [file 41594_2024_1418_MOESM7_ESM.jpg]

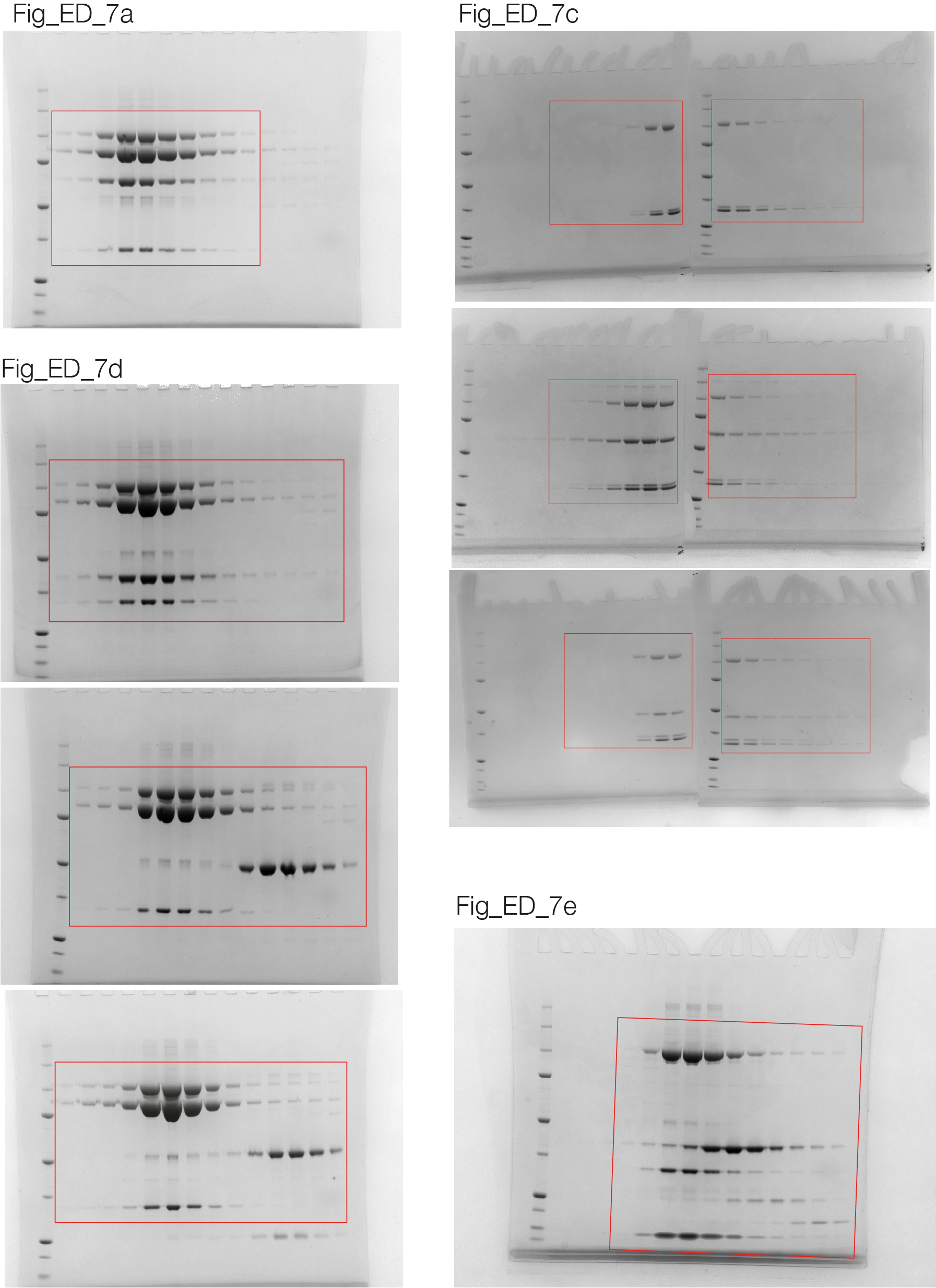

Supplement: Supplementary file 8 — Unprocessed coomassie stained SDS–PAGE gels of Extended Data Fig. 7a,c,d,e. [file 41594_2024_1418_MOESM8_ESM.jpg]
